# Human CD34^+^/CD90^+^ ASCs Are Capable of Growing as Sphere Clusters, Producing High Levels of VEGF and Forming Capillaries

**DOI:** 10.1371/journal.pone.0006537

**Published:** 2009-08-06

**Authors:** Francesco De Francesco, Virginia Tirino, Vincenzo Desiderio, Giuseppe Ferraro, Francesco D'Andrea, Mariateresa Giuliano, Guido Libondi, Giuseppe Pirozzi, Alfredo De Rosa, Gianpaolo Papaccio

**Affiliations:** 1 Dipartimento di Medicina Sperimentale, Sezione di Istologia ed Embriologia, Tissue Engineering and Regenerative Medicine (TERM) Laboratory, Seconda Università di Napoli, Napoli, Italy; 2 Dipartimento di Scienze Ortopediche, Traumatologiche, Riabilitative e Plastico-Ricostruttive, Seconda Università di Napoli, Napoli, Italy; 3 Dipartimento di Medicina Sperimentale, Sezione di Biotecnologie, Seconda Università di Napoli, Napoli, Italy; 4 UOC Biologia cellulare e Bioterapia, Istituto Nazionale Tumori “G. Pascale”, Napoli, Italy; 5 Dipartimento di Scienze Odontostomatologiche, Ortodontiche e Chirurgiche, Seconda Università di Napoli, Napoli, Italy; INSERM, France

## Abstract

**Background:**

Human adult adipose tissue is an abundant source of mesenchymal stem cells (MSCs). Moreover, it is an easily accessible site producing a considerable amount of stem cells.

**Methodology/Principal Findings:**

In this study, we have selected and characterized stem cells within the stromal vascular fraction (SVF) of human adult adipose tissue with the aim of understanding their differentiation capabilities and performance. We have found, within the SVF, different cell populations expressing MSC markers – including CD34, CD90, CD29, CD44, CD105, and CD117 – and endothelial-progenitor-cell markers – including CD34, CD90, CD44, and CD54. Interestingly, CD34^+^/CD90^+^ cells formed sphere clusters, when placed in non-adherent growth conditions. Moreover, they showed a high proliferative capability, a telomerase activity that was significantly higher than that found in differentiated cells, and contained a fraction of cells displaying the phenotype of a side population. When cultured in adipogenic medium, CD34^+^/CD90^+^ quickly differentiated into adipocytes. In addition, they differentiated into endothelial cells (CD31^+^/VEGF^+^/Flk-1^+^) and, when placed in methylcellulose, were capable of forming capillary-like structures producing a high level of VEGF, as substantiated with ELISA tests.

**Conclusions/Significance:**

Our results demonstrate, for the first time, that CD34^+^/CD90^+^ cells of human adipose tissue are capable of forming sphere clusters, when grown in free-floating conditions, and differentiate in endothelial cells that form capillary-like structures in methylcellulose. These cells might be suitable for tissue reconstruction in regenerative medicine, especially when patients need treatments for vascular disease.

## Introduction

Stem cells are undifferentiated cells that undergo multiple, sequential cell divisions, that have the ability to renew themselves, and that, in the adult, are responsible for repair and repopulating damaged tissues [1]. Therefore, they are the natural and direct source from which stable differentiated cells can form an adult tissue. Moreover, they can differentiate into specialized cell types [2]. Mesenchymal stem cells of bone marrow (BM-MSCs) have been studied for years [3], [4]; these cells can differentiate into adipocytes, osteoblasts [5] and other cells of mesenchymal origin [6], [7] . Recently, new sources of adult stem cells have been discovered in different tissues of the human body, including dental pulp [8], [9], Wharton Jelly [10], [11], amniotic membrane [12], and adipose tissue [13].

Adipose tissue represents an important source of adult stem cells, called ASCs (adipose stem cells) [14], [15]. ASCs share many characteristics of bone marrow, including extensive proliferation and the ability to undergo multilineage differentiation [16], and they display a noticeable plasticity both *in vitro* [17] and *in vivo* [18]. In fact, ASCs can express the biochemical profile of adipocytes, chondrocytes, and osteoblasts under appropriate culture conditions *in vitro* [19], [20]. In addition, the stromal vascular fraction (SVF) of human adipose tissue has been recently reported to be capable of differentiating into endothelial cells [21]; it has been also demonstrated that these cells can enhance neovascularization in a murine ischemic model [22] and secrete angiogenic growth factors [23]. Moreover, a large number of stem cells can be obtained from adipose tissue because it is abundant in the body and highly accessible; thus, ASCs are tremendous interest to regenerative medicine [24].

The aim of this study was to characterize stem/progenitor cells found within the SVF of adipose tissue and label their phenotypic profile during the steps of isolation, purification, and expansion. In particular, their kinetics were followed though the expression of different markers for stem and differentiated cells. We have focused our attention on progenitor cells showing both an angiogenic and adipogenic potential.

Our study has shown the following results and novelties with respect to the existing literature on this topic: (i) two different populations can be detected within the SVF of human adipose tissue: mesenchymal stem cells expressing CD90, CD29, CD44, CD105, CD117, and CD34; and endothelial progenitor cells, expressing CD34, CD90, CD44, and CD54; (ii) CD34^+^/CD90^+^ cells are capable of differentiating into endothelial cells (CD31^+^/VEGF^+^/Flk-1^+^) and, when stimulated with an adipogenic medium, into adipocytes; (iii) these cells are also capable of forming sphere clusters, when grown in free-floating conditions, and capillary-like structures, when challenged on methylcellulose, as substantiated by production of VEGF.

## Results

### Cell Culture

After enzymatic digestion of adipose tissue, samples of 1,000,000 cells were collected from the cell suspension and cultured. After three days of culture, adherent cells acquired a fibroblast-like or polygonal shape morphology. This morphology was maintained through several passages under standard culture conditions from cells belonging to both mammary and abdominal regions.

### Flow cytometry assay

**Figure 1:**
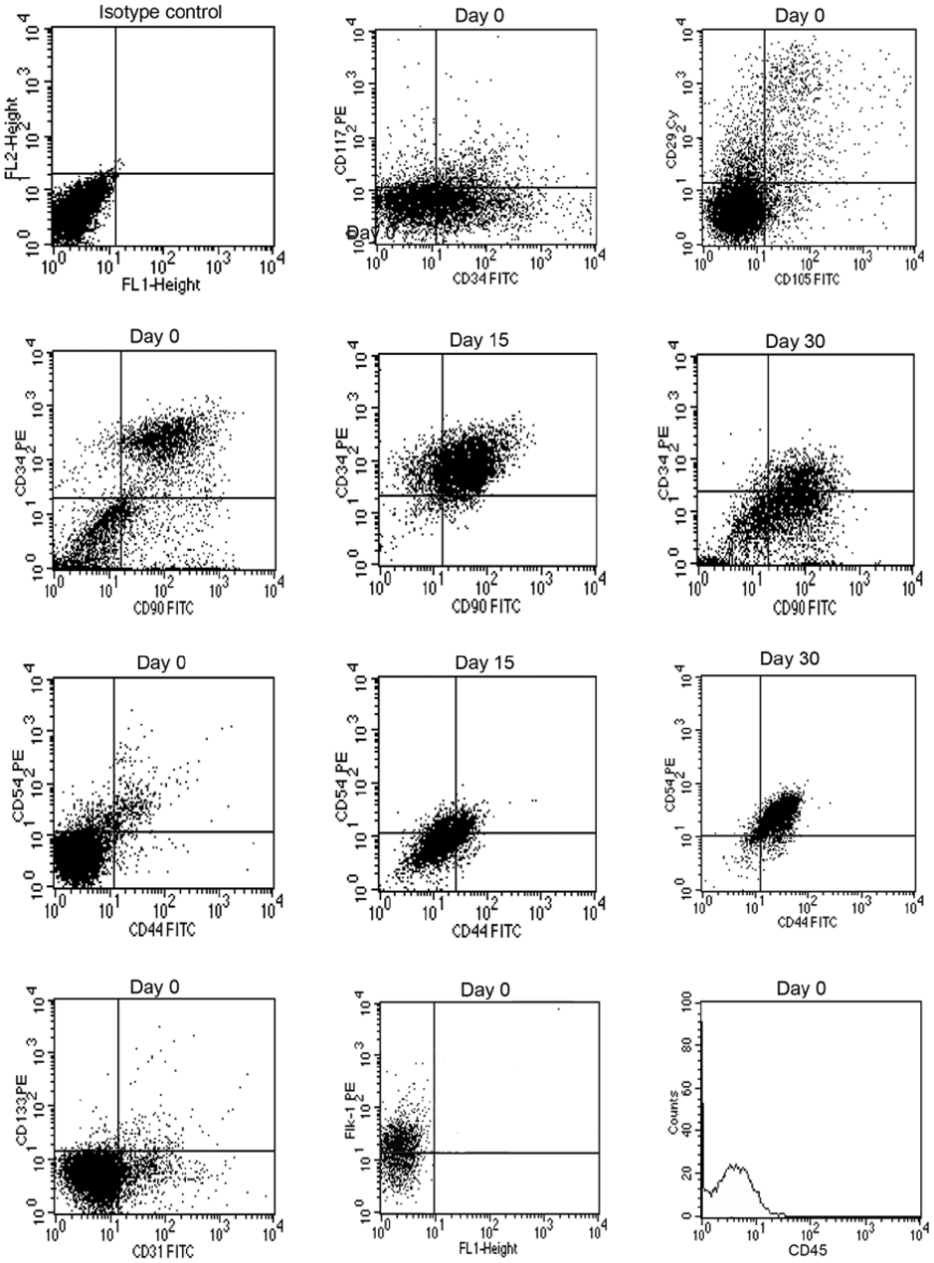
Representative flow cytometry analysis performed at day 0, 15, and 30 of culture. A significant number of ASCs at day 0 are clearly positive for mesenchymal markers, including CD29 (89%), CD105 (12%), CD34 (33%), CD90 (52%), CD117 (10%), and for endothelial markers, including CD31 (9%), CD133 (4%), CD44 (5%), CD54 (9%), and Flk-1 (32%). At day 15 the CD34 and CD90 levels consistently increased (80%). After 30 days of culture, CD34 levels significantly decreased down to 8%, while the CD90 expression held steady at 80%. Later, cells were mainly positive for CD44 and CD54 antigens (87%). Control PE-conjugated and FITC-conjugated isotypes were negative and all cells were negative for CD45 antigens.

**Figure 2:**
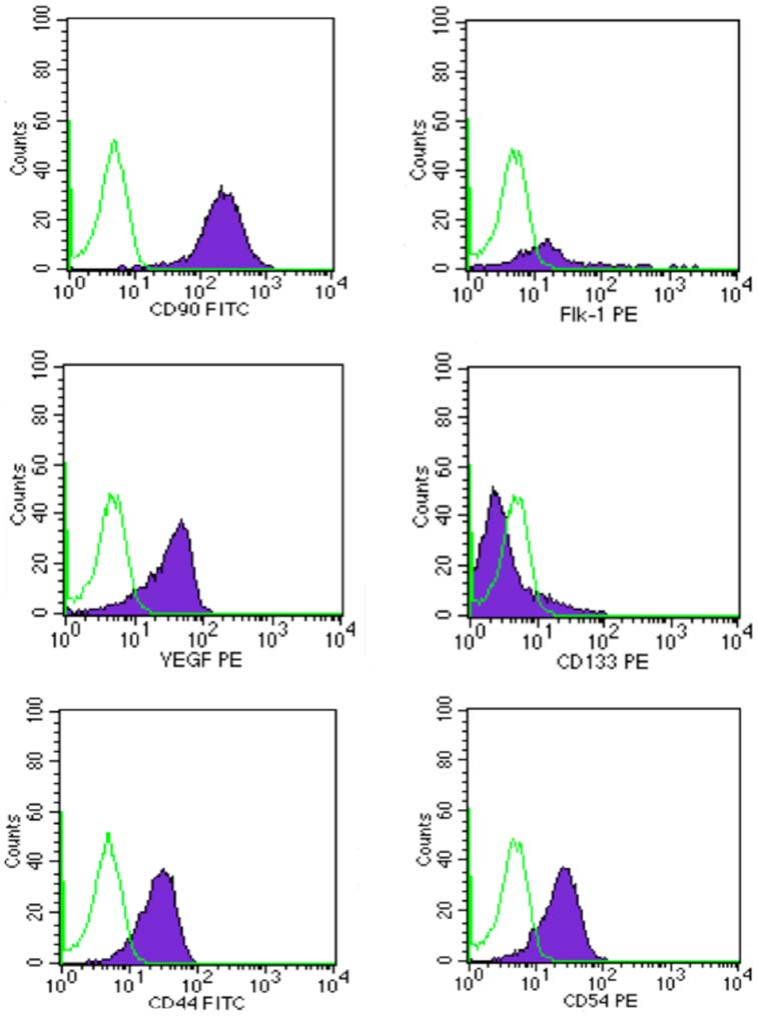
Representative flow cytometry analysis performed on CD34^+^/CD90^+^ cells at day 30 from sorting. A significant number of ASCs are clearly positive for endothelial markers, including CD90 (97%), CD44 (90%), CD54 (90%), VEGF (70%), CD133 (18%), and Flk-1 (60%). Control PE-conjugated and FITC-conjugated isotypes were negative.

At day 0, antigenic analysis of cells belonging to both mammary and abdominal regions showed that different cell types were detectable within the SVF; these were positive for CD29 (β1-integrin) (89%), CD34 (sialomucin and L-selectin ligand) (33%), CD90 (Thy-1) (52%), CD117 (10%), CD105 (endoglin) (12%), and CD133 (4%); and for differentiated cell-associated markers such as CD31 (9%), CD44 (5%) CD54 (9%), and Flk-1 (VEGFR-2) (32%). At day 15, the number of CD34^+^ and CD90^+^ cells belonging to both mammary and abdominal regions increased consistently up to 80%. After 30 days of culture, CD34 decreased significantly down to 8%, while CD90 expression held steady at 80% (Fig. 1). At this time, sorted CD34^+^/CD90^+^ cells acquired an endothelial morphology and expressed high levels of endothelial markers such as CD90 (97%), CD44 (90%), CD54 (90%), VEGF (70%) , CD133 (18%), and Flk-1 (60%) (Fig. 2). All cells tested were negative for CD14 (monocyte marker) and CD45 (leucocyte marker). The absence of these markers distinguished the different cell populations from cells of hematopoietic lineage.

### Proliferation Assay

**Figure 3:**
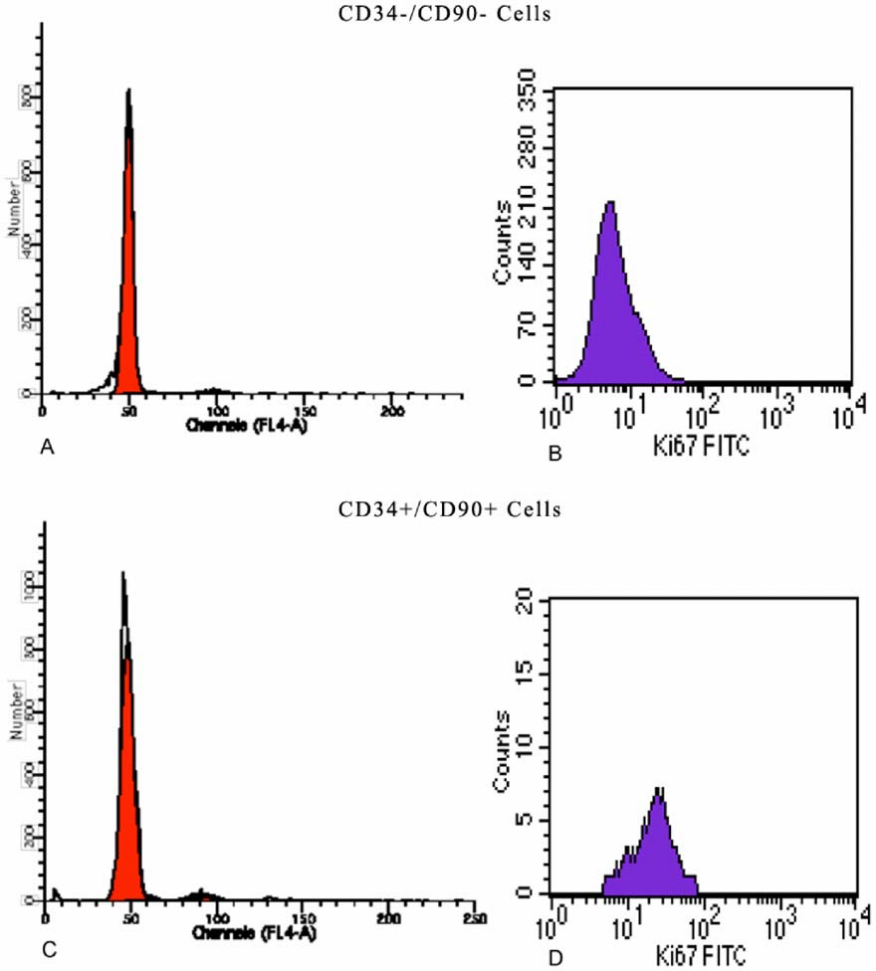
Cell cycle analysis performed using Hoechst 33342 and Ki67 both on CD34 negative and positive cells. (A) Hoechst 33342 analysis performed CD34^−^ cells: G_0_G_1_ phase (98%), S phase (0,65%) and G_2_M phase (0,50%); (B) Ki67 analysis performed CD34- cells: Ki67 (10%); (C) Hoechst 33342 analysis performed CD34^+^ cells: G_0_G_1_ phase (84%), S phase (5%) and G_2_M phase (10%); (D) Ki67 analysis performed CD34+ cells: Ki67 (85%).

Hoechst 33342 analysis performed both on CD34^−^ and CD34^+^ cells showed that CD34^−^ cells were mostly in G0/G1 phase with respect to CD34^+^ cells (Fig. 3A,C). Ki-67 is a nuclear protein expressed only in proliferating cells, and cell cycle events can be detected following Ki-67 expression: in fact, cells in late G1 phase, S phase, and G2/M phase are Ki-67^+^.

We found that all CD34^+^/CD90^+^ cells resulted to be Ki-67^+^, whereas the majority of CD34^−^/CD90^−^ cells were negative for this marker (Fig. 3 B,D). Thus, our results confirm that the CD34^+^/CD90^+^ fraction is the source of stem cells. Moreover, CD34^+^/CD90^+^ cells exhibited a high proliferative capacity in culture; in fact, after 7 days, the proliferation rate was higher (85%) than that of CD34^−^/CD90^−^ cells (10%).

### Side population analysis

**Figure 4:**
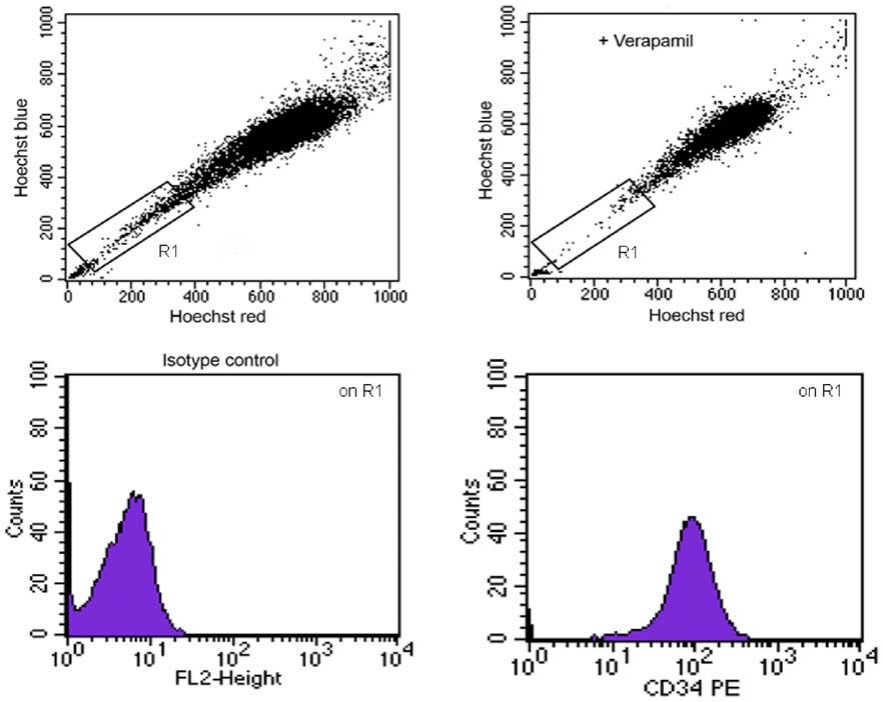
Side population assay. Image of the CD34^+^ fraction representing a very small subset (2%), expressing the characteristic side-population profile.

After enzymatic digestion, the SVF cells of adipose tissue were labeled with Hoechst 33342 to detect the side population (SP). We found that the SVF contains only a small number of cells with SP characteristics (2% of the total population). These cells were all positive for CD34 (Fig. 4).

### Sphere Cluster Formation

**Figure 5:**
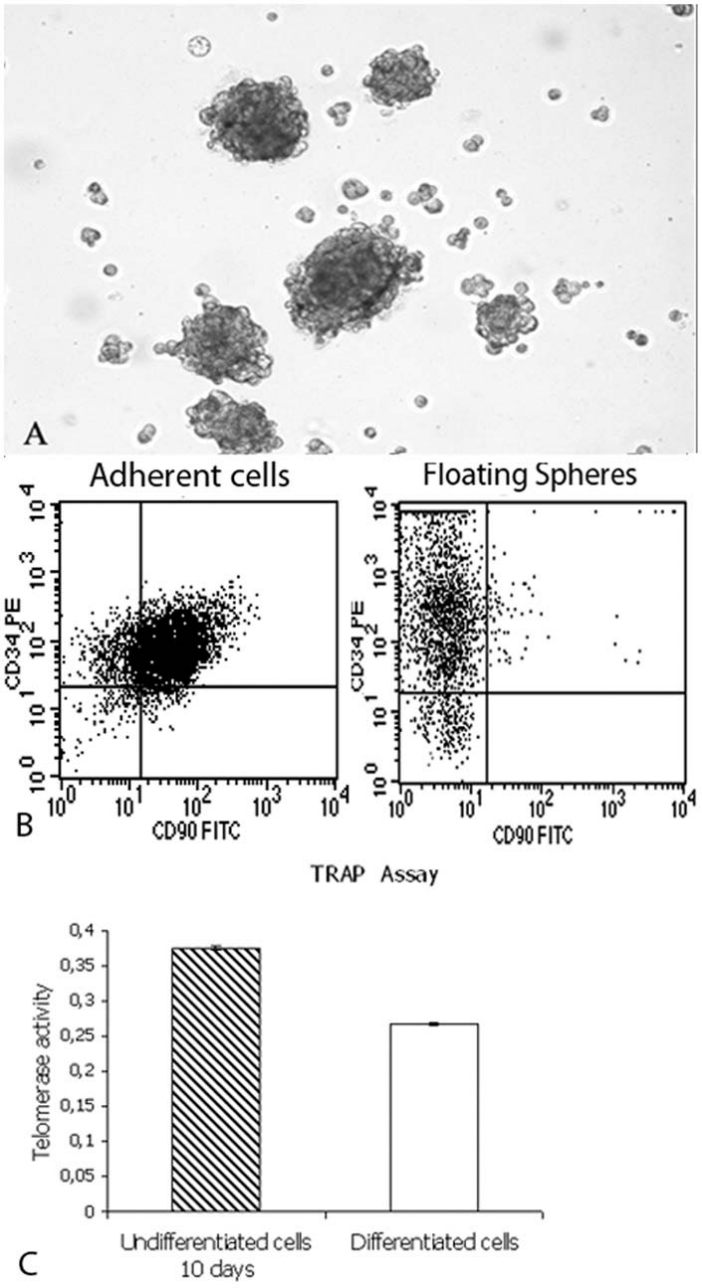
Spheres formation, cytometric analysis and telomerase activity. (A) Sphere clusters formed by CD34^+^/CD90^+^ cells in semisolid medium after 24 hours (Original Magnification×100); (B) Cytometric analysis on adherent cells for CD90 (80%) and CD34 (80%) antigens and on floating spheres for CD90 (2–3%) and CD34 (95–98%) antigens; (C) Telomerase activity of differentiated endothelial cells (ΔA = 0.160) was significantly reduced (p<0.001) respect to undifferentiated CD34^+^/CD90^+^ cells (ΔA = 0.377)

Cultured CD34^+^/CD90^+^ cells formed spheres already after 24 h whereas CD34^−^/CD90^−^ did not (Fig. 5A). After 7 days of culture, CD34^+^/CD90^+^ cell-spheres were seeded into standard medium with 10% FBS. Cells migrated from the spheres within a few hours and adhered to the bottom of the flasks, where they assumed a fibroblast-like shape. Moreover, in order to investigate whether the spheres maintained or increased CD34 expression, we performed analysis for CD34 and CD90 at passage 5. We have found that the spheres become enriched in CD34 (95–98%) with low levels of CD90 (2–3%) (Fig. 5C). These results were observed both in CD34^+^/CD90^+^sorted cells and in non-sorted cells.

### Telomerase activity

The majority of researchers report that differentiation down-regulates telomerase activity both in stem cells and in cancer cell lines. It is also known that telomere shortening occurs during replicative ageing, possibly at a slower rate than in normal somatic cells. Therefore, to establish whether stem cells were induced to differentiate and to undergo replicative senescence, we evaluated telomerase expression. In our hands, at 30 days of culture, telomerase activity of differentiated endothelial cells (ΔA = 0.160) was significantly reduced (p<0.001) with respect to undifferentiated CD34^+^/CD90^+^ cells (ΔA = 0.377) (Fig. 5B).

### In vitro adipogenic differentiation

**Figure 6:**
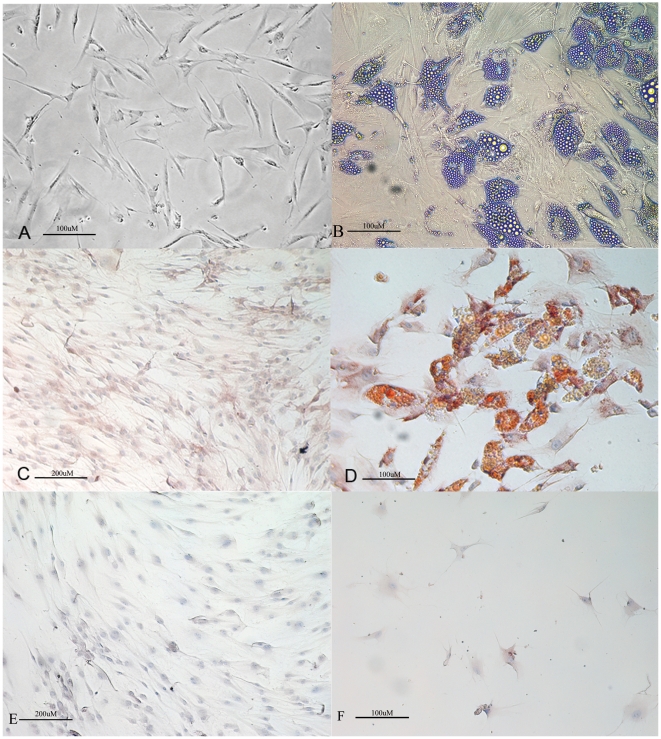
Adipogenic differentiation. (A) ASCs in DMEM 10% FBS exhibit a fibroblast-like morphology (Original magnification 100×); (B) ASCs in adipogenic medium exhibit an adipocyte morphology (Original magnification 100×); (C) CD34^+^/CD90^+^ cells in DMEM 10% FBS showing negativity for adiponectin by immunohistochemistry (Original magnification 400×); (D) CD34^+^/CD90^+^ cells in adipogenic medium showing positivity for adiponectin by immunohistochemistry (Original magnification 100×); (E) CD34^−^/CD90^−^ cells in DMEM 10% FBS showing negativity for adiponectin by immunohistochemistry (Original magnification 100×); (F) CD34^−^/CD90^−^ cells in adipogenic medium showing negativity for adiponectin by immunohistochemistry (Original magnification 400×).

The effect of adipogenic conditions on the cell subtypes of the SVF was studied. Both sorted (CD34^+^/CD90^+^ as well as CD34^−^/CD90^−^) and non-sorted cells were placed in adipogenic medium for three weeks. Under this condition, CD34^+^/CD90^+^ cells acquired the typical morphology of lipid-laden cells containing intracellular lipid droplets (Fig. 6A,B), that were positive at immunoistochemistry for adiponectin already after 24 h (Fig. 6C,D). On the contrary, CD34^−^/CD90^−^ cells were not able to differentiate into adipocytes and they did not express adipogenic markers (Fig. 6E,F).

### In vitro angiogenic differentiation

**Figure 7:**
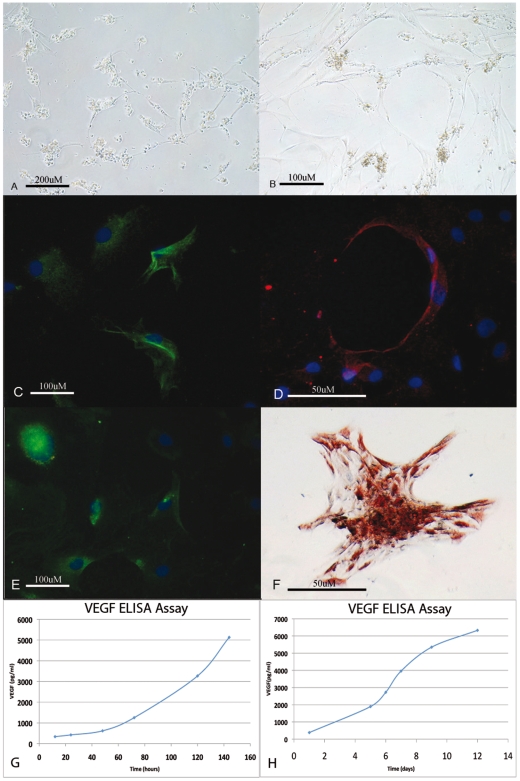
Endothelial differentiation. (A) Image showing differentiated endothelial cells that, after 48 h in methylcellulose, acquire a different morphology comprising round cells with cytoplasmic granules and large flat cells with endothelial morphology (Original magnification 400×); (B) Image showing differentiated endothelial cells that, after day 7 in methylcellulose, formed an extensive intercellular tube networks (Original magnification 400×); Immunofluorescence images of differentiated endothelial cells showing positivity for several specific markers including (C) CD90 (Original magnification 100×); (D) VEGF (Original magnification 100×); (E) CD31 (Original magnification 100×); (F) Immunohistochemistry images of differentiated endothelial cells showing positivity for VEGF (Original magnification 100×); (G) At 140 hours, VEGF quantity was found to be of 5130 pg/ml with respect to the value of 336 pg/ml found at 12 hours; (H) At day 12, VEGF was 6331 pg/ml of medium with respect to 382 pg/ml found at day 1.

The effect of angiogenic conditions was also studied. At 48 h/72 h, CD34^+^/CD90^+^ cells cultured on methylcellulose either with or without VEGF started to form a vascular network (Fig. 7A). After day 7, these cells formed an extensive intercellular tube network including small and large round cells and large flat cells with endothelial morphology that formed clusters (Fig. 7B); most were strongly positive for endothelial markers, including CD90 (Fig. 7C), VEGF (Fig. 7D), CD31 (Fig. 7E), but negative for CD34. The positivity for VEGF antigens was also confirmed by immunohistochemical staining (Fig. 7F).

### ELISA analysis for VEGF

In order to substantiate CD34^+^/CD90^+^ ASCs endothelial differentiation, we analyzed the quantity of VEGF produced by these sorted cells in the culture period at different time points. Results showed that CD34^+^/CD90^+^ ASCs secreted high VEGF levels, which increased with time. In fact, at 140 hours, VEGF quantity was found to be of 5130 pg/ml with respect to the value of 336 pg/ml found at 12 hours, demonstrating a 15-fold increased level (Fig. 7G) with p<0.05. Moreover, at day 12, VEGF was 6331 pg/ml of medium with respect to 382 pg/ml found at day 1, showing a 16-fold increase (Fig. 7H) with p<0.05.

### RT-PCR analysis

**Figure 8:**
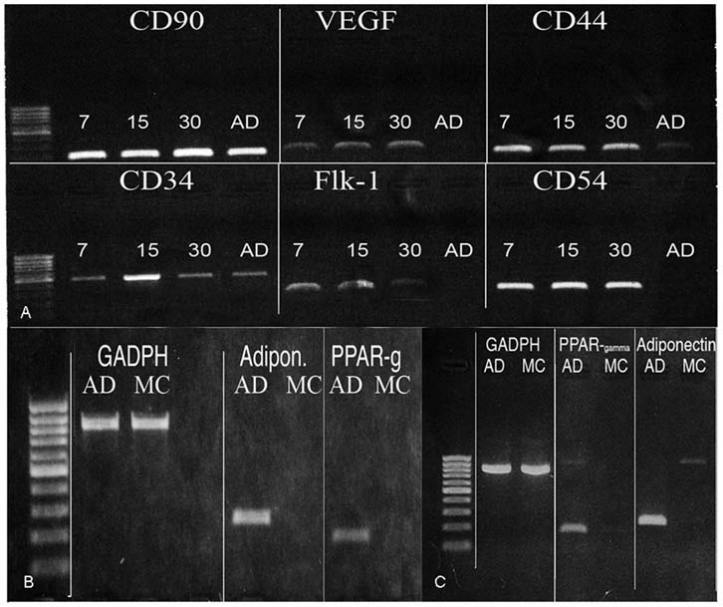
RT-PCR analysis. (A) Representative figure of RT-PCR showing mRNA transcript expression of CD90, CD34, CD44, CD54, VEGF, Flk-1, on cells in DMEM 10% FBS 7, 15 and 30 days of culture; (B) PPARγ and adiponectin on cells in adipogenic medium at 7 and (C) 30 days of culture.

Expression of CD34, CD90, CD44, CD54, VEGF, and Flk-1 mRNA levels, confirmed the results obtained with flow cytometry and immunofluorescence analyses (Fig. 8A).

RT-PCR analyses were performed at 7 and 30 days after sorting on CD34^+^/CD90^+^ cells. The increased lipid storage within the CD34^+^/CD90^+^ cells grown under adipogenic condition was characterized by the presence of transcriptional regulators of adipogenesis: the expression of early and late markers of the adipocyte differentiation process was observed, with PPARγ and adiponectin at 7 and 30 days. (Fig. 8B,C).

## Discussion

In this study, we have isolated and characterized stem/progenitor cells from human adult adipose tissue withdrawn from mammary (via lipectomy) and abdominal (via lipectomy and liposuction) regions. We did not observe significant differences between the two sites of withdrawal. Our attention was focused on the stromal vascular fraction (SVF) of adipose tissue. In freshly isolated SVF cells, fluorescence activated cell sorting analyses evidenced the presence of two cell subpopulations: a CD34^+^/CD90^+^/CD29^+^/CD44^+^/CD105^+^/CD14^−^/CD45^−^ mesenchymal stem/progenitor cell population; and a CD44^+^/CD54^+^/VEGF^+^/Flk-1^+^ endothelial cell population. We decided to study the cell subset co-expressing CD34 and CD90 antigens, and evaluated surface marker expression at 0, 15, and 30 days from fresh tissue samples – this because the CD34 marker is an antigen identified on the surface of hematopoietic stem cells, but it is well expressed also on stromal stem cells, fibroblasts, hematopoietic progenitors [25], and endothelial progenitors [26]. Analysis of CD34 with other markers such as CD14 and CD45 (hematopoietic progenitors); CD90, CD117, and CD105 (mesenchymal stem cells); and CD31 and CD133 (endothelial progenitors) is an useful tool for stem-progenitor cell characterization. CD90, on the other hand, is generally expressed on thymocytes, neurons, mesenchymal stem cells, hematopoietic stem cells, NK cells, endothelium (mainly in high endothelial venules (HEVs), where diapedesis takes place, renal glomerular mesangial cells, circulating metastatic melanoma cells, follicular dendritic cells (FDC), a fraction of fibroblasts, and myofibroblasts.

Our study has revealed a significant expression of both CD34 and CD90 markers in cells freshly isolated from the adipose SVF. The absence of CD14 and CD45 distinguished this cell subset from cells of hematopoietic lineage. During cultures, the CD34 antigen underwent down-regulation, whereas CD90 expression held steady while cells acquired the following antigens: VEGF^+^, CD44^+^, CD54^+^, and Flk-1^+^, confirming the endothelial differentiation.

The novelty of this study lives in the demonstration that, in the SVF, there is a CD34^+^/CD90^+^ stem cell population capable of differentiating into endothelial cells and forming capillary-like structures in a semisolid medium with or without the use of angiogenic factors. In fact, during the first 12 h of culture, we have found that the non-adherent cells, grown in methylcellulose, with or without the addition of VEGF, formed small-sized, large-sized round cells with cytoplasmic granules and larger flat cells with an endothelial morphology. After 24 h, cell clusters increased in size and formed connections among them, whereas after 48 h/72 h, cells with an endothelial morphology, formed an extended intercellular tubular network. On the other hand, this population, when isolated at day 0 by flow cytometry, gave rise to multivacuolar adipocytes, if cultured in adipogenic medium. In addition, these cells are capable of forming sphere clusters in a serum-free medium supplemented with bFGF and EGF. Interestingly, during the time of floating culture, spheres maintained the expression of the CD34 antigen, whereas CD90 levels became down-regulated. This was observable either in the case of the CD34/CD90 sorted cell population or in non-sorted cells.

The finding that floating cell cultures maintain and even increase the stemness potential of stem/progenitor-derived cells, probably inhibiting differentiation patterns, must be regarded as another significant result of this study. Consequently, this technique may be used to maintain stem characteristics for a longer time with respect to traditional adhesion cultures. In fact, our CD34^+^/CD90^+^ stem cells differentiated in to endothelial cells after 30 days from cell sorting in adhesion cultures, whereas they maintained their stemness up to 60 days, when cultured as floating sphere clusters.

Telomerase activity of differentiated cells was also significantly reduced with respect to the parental CD34^+^/CD90^+^ cell subset. Telomeres are specific structures found at the end of chromosomes in eukaryotes. In human chromosomes, telomeres consist of thousands of copies of 6 base repeats (TTAGGG). It has been suggested that telomeres protect chromosome ending, because damaged chromosomes lacking telomeres undergo fusion, rearrangement, and translocation. In somatic cells, telomere length is progressively shortened after each cell division both *in vivo* and *in vitro*, due to the inability of the DNA polymerase complex to synthesize the 5′ end of the lagging strand.

Moreover, CD34^+^CD90^+^ cells had a high proliferative potential, and a small fraction of CD34^+^ cells (2%) expressed the characteristic profile of a side population. It is known that the side-population phenotype is the most significant attribute of stem cells. This study is the first to report the presence of a side population in the SVF of adipose tissue. We hypothesize that this side population is formed of primitive stem cells.

Taken together, our data show that co-expression of CD34 and CD90 antigens identifies a cell population capable of forming vessels. This might have an important impact on therapeutic strategies for ischemic tissue damage of myocardium, retina, and limbs [22], [27] because these cells secrete angiogenic cytokines such as IL-1β, which plays a crucial role in the angiogenic process [28].

Recent studies indicate that portions of ischemic and tumor neovasculature are due to a “neovasculogenesis”, whereby bone marrow (BM)-derived circulating endothelial progenitor cells (EPCs) home to sites of regenerative or malignant growth and contribute to blood vessel formation. Therapeutic angiogenesis is an important survival mechanism that preserves the integrity of tissues subjected to ischemia [29], [30].

Currently, autologous endothelial progenitor cells (EPCs) can be isolated and expanded from adult human peripheral blood for therapeutic angiogenesis [31] . However, the number of EPCs obtained from peripheral blood is limited. At present, it is difficult to use autologous cord blood-derived EPCs clinically, therefore, other sources of EPCs should be explored.

In this study, we have demonstrated that our cells gradually lose their positivity for CD34 and acquire an endothelial morphology, expressing high levels of endothelial markers, including CD31, CD44, CD54, Flk1, and VEGF. Moreover, as substantiated by the ELISA assay, VEGF was produced at very high levels in the standard medium during culture. Therefore, our CD34^+^/CD90^+^ cells seem to be different from those reported by Cao et al. [32] which expressed Flk1, but not CD34. Moreover, a large amount of studies indicate that VEGF is required and necessary to promote endothelial differentiation starting from mesenchymal stem cells. In this study, we have demonstrated that CD34^+^/CD90^+^ cells of human adipose tissue differentiate in endothelial cells with formation of capillary-like structures in methylcellulose due to their own VEGF production. In addition, we have demonstrated that it is possible to maintain stemness for a long time during cell culture through sphere growth. These findings suggest that stem cells isolated from SVF of human adipose tissue are involved in angiogenesis in order to repair vascular damage and in forming new blood vessels. Moreover, as previously demonstrated by us [24], vessel formation from ASCs is of high interest because this is involved in the genesis of a tissue *in vitro*.

In conclusion, this study has shown the following: (i) within human adipose tissue of subjects of 18 to 45 years of age, a rather consistent number of CD34^+^/CD90^+^/CD45^−^ stem cells can be selected; (ii) these cells can be considered mesenchymal stem cells and are able to differentiate into the same different lineages characteristic of MSCs: in fact, both stromal and mesenchymal stem cells become progressively CD34^−^ while differentiating and this excludes contamination from marrow fibroblasts; (iii) in culture, these cells proliferate and differentiate on methylcellulose into endotheliocytes due to their high production levels of VEGF, and, under specific stimuli, into multivacuolar adipocytes, as confirmed by RT-PCR for adiponectin and PPAR-γ. The strong PPARγ expression suggests that this gene has a predominant role during cell differentiation; (iv) in free floating cultures, these cells are capable of forming sphere clusters, which can preserve cell stemness for a long period; and (v) they have high telomerase activity, leading to a long lifespan. Moreover, ASCs can be frozen in liquid nitrogen for at least one year without loss of their stemness [33].

Thus, stem cells isolated from the SVF of human adult adipose tissue are an easily accessible, potentially abundant source of cells suitable for clinical applications in vascular regenerative medicine and in soft tissue engineering.

## Materials and Methods

### Adipose tissue harvesting and digestion

Subcutaneous adipose tissue from abdomen and mammary was obtained following written informed consent, approved by our Internal Ethical Committee (Second University Ethical Committee) from 54 female patients with a mean age of 35±0,8 years and with a mean BMI of 26±1.1 Kg/m^2^ that had endured elective procedures for plastic surgery. Adipose tissue was obtained by lipectomy or liposuction in the Plastic and Reconstructive Surgery Clinic of the Second University of Naples. The adipose tissue was placed in a physiological solution (0.9% NaCl), washed twice in PBS (phosphate saline buffer: 137 mM NaCl, 2.7 mM KCl, 10 mM Na_2_HPO_4_, 1.8 mM KH_2_PO_4_), scraped, and placed in a digestion solution: collagenase type I (3 mg/ml) and dispase (4 mg/ml) supplemented with penicillin (100 U/ml), streptomycin (100 µg/ml), and clarythromicin (500 µg/ml) in PBS at 37°C in agitation for 60 min. The digest was filtered through 70 µm filters (Becton & Dickinson, Sunnyvale, CA).

### Cell culture

After filtration and washing, the pellet was resuspended in erythrocyte lysis buffer (155 mM NH_4_Cl, 10 mM KHCO_3_, 0.1 mM EDTA, pH 7.3) for 10 min at room temperature. The cell suspension was centrifuged at 1300 rpm for 7 min and the pellet resuspended in 5 ml Dulbecco's modified Eagle's medium (DMEM) with 10% fetal bovine serum (FBS), 2 mM L-glutamine, 100 U/ml penicillin, and 100 µg/ml streptomycin and seeded in 25 cm^2^ flasks. Flasks were incubated at 37°C under 5% CO_2_ and the medium changed twice a week. Cells reached confluence in 5–7 days. Experiments were performed in quadruplicate.

### Flow cytometry

Cells were detached using trypsin-EDTA (200 mg/L EDTA, 500 mg/L trypsin; Cambrex). At least 200,000 cells were incubated with primary antibody for 30 min at 4°C, washed twice in PBS, and incubated with a secondary antibody. Alternatively, cells were incubated directly with fluorescent-conjugated antibodies for 30 min at 4°C, washed, and resuspended in 0.6 ml PBS. Samples were analyzed at day 0 (day of surgery), day 15, and day 30 by flow cytometry using a FACS Vantage cell sorter (Becton & Dickinson, Mountain View, CA,USA). The antibodies used in this study were: anti-CD117 PE (c-kit) (Miltenyi–Biotech, Calderara di Reno, Bologna, Italy); anti-CD34 FITC and PE (Miltenyi-Biotech); anti-CD90 FITC (BD Pharmingen, Buccinasco, Milano, Italy); anti-CD105 FITC (Santa Cruz, CA, USA); anti-CD29 Cy (Miltenyi-Biotech); anti-CD31 FITC (Miltenyi-Biotech); anti-CD133 PE (Miltenyi-Biotech); anti-hVEGF (Santa Cruz, CA); anti-VEGFR-2 (Santa Cruz); anti-CD54 PE (Miltenyi-Biotech); anti-CD44 FITC (Miltenyi-Biotech), anti-CD45 Cy and PE (BD Pharmingen, Buccinasco, Milano, Italy); and anti-CD14 PE (Miltenyi-Biotech).

For intracellular staining of Ki67 (Miltenyi-Biotech), CD34^+^/CD90^+^ cells were processed using the Caltag Fix & Perm Kit (Invitrogen, Milan, Italy) following the manufacturer's guidelines. All data were analyzed using CellQuest software (Becton & Dickinson, Mountain View, CA,USA).

For DNA staining , cells were incubated for 90 min at 37°C in basal medium with Hoechst 33342 (5 µg/ml Sigma, Milan, Italy). Data analysis was performed with ModFit 3.0 software.

### Cell Sorting

At day 0, CD34^+^/CD90^+^ cells were sorted and cultured in order to perform the following experiments: sphere assay, differentiation assays, proliferation assay, side population assay, and telomerase activity. This isolated CD34^+^/CD90^+^ population expresses neither the leukocyte marker, CD45, nor the monocyte/macrophage marker, CD14. Both sorted CD34^−^/CD90^−^ and non-sorted cells were used as controls.

### Side Population Analysis

After enzymatic digestion, aliquots of SVF cells were washed with ice-cold PBS/2% FBS. Cells (500,000) were labeled in the growth medium with 5.0 µg/ml Hoechst 33342 dye, either alone or in combination with 50 µg/ml verapamil at 37°C for 90 min. After washing with PBS/2% FBS, the cells were incubated with 2 µg/ml propidium iodide to exclude dead cells. The Hoechst dye was excited with UV laser and its fluorescence measured with 675/20 (Hoechst Red) and 424/44 (Hoechst Blue) filters.

### Limited dilution assay and proliferative potential

In order to assess clonogenic as well as proliferative ability of cells, suspensions of stem cells (CD34^+^/CD90^+^/CD45^−^) were diluted and single cells seeded in 96 multiwells. Two weeks later, cells were stained with Toluidine blue in order to assess their proliferation rate.

### Sphere cluster formation assay

Cells were plated at a density of 60,000 cells/well in 6-well ultra low attachment plates (Corning Inc., Corning, NY, USA) in DMEM/F12 cell medium supplemented with 1% methylcellulose, progesterone (10 nM), putrescine (50 µM), sodium selenite (15 nM), transferrin (13 µg/ml), insulin (10 µg/ml; Sigma, Milan, Italy), human EGF (20 ng/ml), and human bFGF (20 ng/ml; Sigma). Fresh aliquots of EGF and bFGF were added every other day. After culture for 48–72 hours, spheres were visible at the inverted phase-contrast microscope (Nikon TS 100, Nikon, Florence, Italy). Moreover, in order to investigate if the spheres may maintain or enrich CD34 expression during suspension culture, we performed CD34 and CD90 analysis at passage 5, starting both from cells sorted for CD34/CD90 and the total cell population. The spheres were detached using trypsin-EDTA (200 mg/L EDTA, 500 mg/L trypsin; Cambrex) and analyzed for CD34PE (Miltenyi-Biotech) and CD90 FITC (BD Pharmingen) by flow cytometry.

### Telomerase activity

The TRAPeze ELISA Telomerase Detection Kit was used to perform the telomeric repeat amplification protocol (TRAP) and ELISA assay for the non-quantitative detection of telomerase activity in treated cells versus control, according to manufacturer's instructions (Chemicon International, Milan, Italy). Briefly, the kit is based on two main steps: TRAP extension/amplification and detection. The TRAP extension/amplification reaction is performed with biotinylated primer and a deoxynucleotide mix containing dCTP labeled with dinitrophenyl (DNP). The labeled products are then detected by anti-DNP antibody conjugated to horseradish peroxidase (HRP). The amount is determined by the HRP activity using the substrate, 3,3′,5,5′-tetramethylbenzidine, and subsequent color development. Exponentially growing cells were seeded into 75 cm^3^ flasks at 2×10^5^ cells/mL in triplicate. Differentiation was induced up to day 21. Total proteins of cell extracts were quantified using the BioRad assay. The positive control was provided by the supplier of the kit. For the negative control, extracts from cells were heated at 65°C for 10 min. The protein extract was considered telomerase-positive if the net increase of absorbance for the sample ΔA (A_sample_−A_heat-treated sample_) was>0.150.

### Adipogenic differentiation

Cells were induced in the following adipogenic medium for 2–3 weeks: DMEM supplemented with 10% FBS plus dexamethasone (1 µM; Sigma), human recombinant insulin (10 µM; Sigma), indomethacin (200 µM; Fluka, Milan, Italy) and 3-isobutyl-1-methyl-xantine (IBMX) (0.5 mM; Sigma). Cells cultured in basal medium (see above) were used as controls.

### Angiogenic differentiation using methylcellulose

To analyze in vitro capillary-like morphology, 2×10^5^−5×10^5^ cells/ml were plated in 24-well plates in a semisolid growth medium that consisted of 0.9% methylcellulose in DMEM, 30% FBS, 1% bovine serum albumin (BSA), 10^−4^ mol/L mercaptoethanol, and 2 mmol/L L-glutammine. In parallel experiments, cultures were stimulated in addition with vascular endothelial growth factor (VEGF, 50 ng/ml). All cultures were performed in triplicate, incubated at 37°C under 5% CO_2_ and left for 7 days to develop a capillary-like morphology.

### ELISA assays

In order to quantify VEGF levels produced in the standard medium from cells CD34^+^/CD90^+^, we performed VEGF ELISA assay with an Endogen detection kit (TEMA research, Bologna, Italy) according to the manufacturer's protocol. The standard mediums were collected at 1, 5, 6, 7, 9, 12 days in the first assay and at 12, 24, 48, 72, 120 and 144 hours from sorting in the second assay.

### Immunofluorescence and Immunohistochemical staining

Cells in P6 well plates were washed in PBS and fixed with 4% PFA for 30 min at 4°C, then washed three times in PBS for 10 min and incubated in PBS/5%FBS for 60 min at 4°C. After a double washing in PBS for 10 min at room temperature, cells were incubated overnight at 4°C with monoclonal anti-human antibodies (diluted 1∶100 in PBS). Wells were washed in PBS three times for 10 min at room temperature and incubated for 90 min at 4°C with the secondary FITC- or PE-conjugated antibody (diluted 1∶200 in PBS 1X) (Santa Cruz, CA, USA). Moreover, cells were stained with DAPI (4′, 6-diamidino-2-phenylindole) (Invitrogen, San Giuliano Milanese, Milan, Italy) diluted 1∶10000 (5 µg/ml) in PBS for 7 min at room temperature. Cells incubated for 90 min at 4°C only with conjugated secondary antibodies were used as negative control. Cells were then observed under a fluorescence microscope (Nikon Instruments Italia, Calenzano, Firenze, Italy). Primary antibodies included: anti-CD31, anti-CD90 and anti-VEGF, all purchased from Santa Cruz, CA, USA.

Immunohistochemical analyses were performed with a DAKO CYTOMATION kit (En Vision + System-HRP-AEC, Dako Italia, Milan, Italy) according to the manufacturer's protocol. Antibodies used were: anti-adiponectin and anti-VEGF, all purchased from AbCam, Cambridge, UK.

### RNA isolation and polymerase chain reaction

RNA was extracted with TRI Reagent (Sigma, Milan, Italy). cDNA synthesis was lead on total RNA by SuperScript II reverse transcriptase (Invitrogen, San Giuliano Milanese, Milan, Italy). Used primers sequences: *GADPH: fw AGCCGCATCTTCTTTTGCGTC; rw TCATATTTGGCAGGTTTTTCT; CD34: fw AAAGACCCTGATTGCACTGG rw GCCCTGAGTCAATTTCACTT; CD90: fw CCCAGTGAAGATGCAGGTTT; rw GACAGCCTGAGAGGGTCTTG; CD44: fw TCCAAAGGTTTTCCATCCTG; rw AGGGCCAGCCTCTATGAAAT; CD54: fw CAGGTTGTAACACTGCAGGAGAG; rw ATTGTGAACACTGGCAGAAATG; VEGF: fw TGACAGGGAAGAGGAGGAGA; rw CGTCTGACCTGGGGTAGAGA; PPARγ: fw ACAGCAAACCCCTATTCCATGC; rw: ATTACGGAGAGATCCACGGAGC; Adiponectin: fw CAACATTCCTGGGCTGTACT; rw CCTGTGAAGGTGGAGTCATT.*

### Statistical analysis

Student t-test (two-tailed) was used for statistical evaluation. Level of significance was set at p<0.05.
